# Assessment of hospitalisation costs and their determinants among Covid-19 patients in South Central Ethiopia

**DOI:** 10.1186/s12913-023-09988-2

**Published:** 2023-09-04

**Authors:** Abdene Weya Kaso, Esmael Mohammed, Gebi Agero, Gemechu Churiso, Taha Kaso, Helen Ali Ewune, Alemayehu Hailu

**Affiliations:** 1 Department of Public Health, College of Health Science, Arsi University, Assela, Ethiopia; 2 Bokoji Primary Hospital, Oromia Health Bureau, Bokoji, Ethiopia; 3 Department of Medical Laboratory, College of Medicine and Health Science, Dilla University, Dila, Ethiopia; 4 Department of Surgery, College of Health Science, Arsi University, Assela, Ethiopia; 5 Department of Public Health, College of Medicine and Health Science, Dilla University, Dila, Ethiopia; 6 Faculty of Health and Social Science, Section for Global Health and Rehabilitation, Western Norway University of Applied Sciences, Bergen, Norway; 7 Bergen Centre for Ethics and Priority Setting, Department of Global Public Health and Primary Care, University of Bergen, Bergen, Norway

**Keywords:** Covid-19, Cost, Hospitalisation, Determinants

## Abstract

**Background:**

The coronavirus disease 2019 (Covid-19) pandemic is a global public health problem. The Covid-19 pandemic has had a substantial impact on the economy of developing countries, including Ethiopia.This study aimed to determine the hospitalisation costs of Covid-19 and the factors associated with the high cost of hospitalisation in South Central Ethiopia.

**Methods:**

A retrospective cost analysis of Covid-19 patients hospitalised between July 2020 and July 2021 at Bokoji Hospital Covid-19 Treatment Centre was conducted using both the micro-costing and top-down approaches from the health system perspective. This analysis used cost data obtained from administrative reports, the financial reports of the treatment centre, procurement invoices and the Covid-19 standard treatment guidelines. The Student’s t-test, Mann-Whitney U test or Kruskal-Wallis test was employed to test the difference between sociodemographic and clinical factors when appropriate.To identify the determinants of cost drivers in the study population, a generalised linear model with gamma distribution and log link with a stepwise algorithm were used.

**Results:**

A total of 692 Covid-19 patients were included in the costing analysis. In this study, the mean cost of Covid-19–infected patients with no symptoms was US$1,073.86, with mild symptoms US$1,100.74, with moderate symptoms US$1,394.74 and in severe–critically ill condition US$1,708.05.The overall mean cost was US$1,382.50(95% CI: 1,360.60–1,404.40) per treated episode.The highest mean cost was observed for personnel, accounting for 64.0% of the overall cost. Older age, pre-existing diseases, advanced disease severity at admission, admission to the intensive care unit, prolonged stay on treatment and intranasal oxygen support were strongly associated with higher costs.

**Conclusions:**

This study found that the clinical management of Covid-19 patients incurred significant expenses to the health system. Factors such as older age, disease severity, presence of comorbidities, use of inhalation oxygen therapy and prolonged hospital stay were associated with higher hospitalisation costs.Therefore, the government should give priority to the elderly and those with comorbidities in the provision of vaccination to reduce the financial burden on health facilities and health systems in terms of resource utilisation.

## Introduction

Human coronaviruses were first identified in the mid-1960s. They cause mild to moderate upperrespiratory tract illnesses [1, 2]. The novel coronavirus that causes severe acute respiratory syndrome coronavirus 2 (SARS-CoV2) was identified in Wuhan, China in December 2019 [3]. The World Health Organization (WHO) declared the SARS-CoV2 disease a global pandemic and public health emergency after it affected many countries [4]. Up to July 2021, globally, 183,198,019 confirmed cases were reported to WHO, with around 3,971,687 deaths [5]. Ethiopia reported its first Covid-19 case on 13 March 2020 and had reported 162,954 cases and 2,394 deaths due to the pandemic through 1 July 2021 [6, 7].

The occurrence of the Covid-19 pandemic has had a substantial economic impact on the education, health, socioeconomic and tourism activities of many developing countries, including Ethiopia [8–10]. Thus, countries have implemented Covid-19 preventive measures such as school and university closures, obligatory quarantine and restriction of public mass gatherings during meetings and religious festivals to decrease the spread of the virus [11–13]. In addition, countries have implemented the WHO preventive measure recommendations, such as maintaining physical distancing, wearing a face mask and promoting hand washing with soap or hand sanitiser in all service provision areas to reduce the burden of the pandemic [14, 15].

However, disparities in communities’ adherence and belief in the protectiveness of the preventive measures as a whole were a challenge to tackling Covid-19, which can exacerbate the spread of the pandemic [16]. This introduced a multitude of challenges to healthcare systems and health institutions in many countries at all stages of the pandemic due to the surge of Covid-19 cases [17]. For instance, to confront the challenges of Covid-19, hospitals and health institutions in the USA took heroic and unprecedented healthcare measures, such as testing and treating hundreds of thousands of people as outbreaks expanded across the nation, which required billions of dollars [8, 17, 18]. In addition, the healthcare responses, such as the quarantine system, treatment and emergency support for SARS-CoV-2 patients, facilities for infectious disease treatment and medical supplies to stop Covid-19 infection were anticipated to cost about US$52 billion per four weeks in the USA [18, 19]. Additionally, a report from an Iranian hospital revealed that the hospital’s revenue declined by 9%, its costs increased by 70% and the hospital’s balance was reported to be −$607,143 [20]. Furthermore, African economies were reduced by about 1.4% in gross domestic product (GDP) and faced a contraction of up to 7.8% due to the pandemic, which reduced the capacity of governments to extend the public services necessary to respond to the crisis [9, 21]. Furthermore, poor healthcare systems, lack of skilled healthcare professionals, low awareness of disease control and prevention, scarce intensive care units (ICUs) and mechanical ventilators and a high prevalence of underlying diseases were challenges in controlling and preventing the pandemic in low- and middle-income countries, including Ethiopia [21–23].

Despite resource constraints, the Ethiopian government established temporary Covid-19 treatment centres and quarantine centres and covered the costs of the Covid-19 pandemic response,such as mandatory quarantine, contact tracing, testing and the treatment of infected patients, by mobilising resources from domestic and international donors [11, 12]. However, the response to fight against a pandemic brings a substantial challenge due to the competition of health services for limited healthcare resources, as the government must provide other routine and essential health services for communities [23, 24].

Thus, understanding the hospitalisation costs of Covid-19 patients is essential to evaluating the economic impact of the pandemic on healthcare, providing important information for preparedness against the pandemic,planning for future risks and improving knowledge regarding the economic evaluation of global health emergencies. Since a significant number of patients with other health problems require healthcare services [24], an economic evaluation from the hospital’s perspective is also crucial to assess the economic impact of the Covid-19 outbreak on the healthcare system [13].

Therefore, to inform health policy decision-making and resource mobilisation in Ethiopia, it is crucial to comprehend and quantify the direct economic impact of managing hospitalised Covid-19 patients. However, the few studies that identified the costs of treating Covid-19 patients in Ethiopia have not assessed the determinants of hospitalisation costs.Therefore, we intended to determine the costs of Covid-19 hospitalisation and their determinants at Bokoji Hospital Covid-19 Treatment Centre, South Central Ethiopia.

## Methods

### Study setting, design and population

We conducted a facility-based retrospective cross-sectional study in Bokoji Hospital Covid-19 Treatment Centre, which provided services for Covid-19 patients from 28 districts and two town administrations. The treatment centre is situated 175 km from Addis Ababa, the capital of Ethiopia, and 56 km from Asella, a zonal town. This treatment centre has a total of 100 beds, 10 of which are dedicated to ICU patients. To combat the rapid surge of cases in the Arsi zone, Bokoji Primary Hospital was established as a temporary treatment centre to treat Covid-19 patients, and it initiated services on July 1,2020. The treatment centreoperated under the Covid-19 protocol for case management established by the Ethiopian Public Health Institute and Ministry of Health. Patients admitted to the centre with Covid-19 cases were discharged from treatment when they were cured or clinically improved according to the National Covid-19 Treatment and Management Protocol. All Covid-19 patients hospitalised at the treatment centre from July 2020 through July 2021 were included in the study.

### Data collection and analysis

We reviewed the medical records of hospitalised patients retrospectively, and relevant data were extracted and collected by two BSc nurse professionals after a one-day training on the data abstraction form. Data abstraction forms were used to collect patient sociodemographic characteristics (age, sex and residence) and clinical characteristics (status at admission, comorbidities, length of hospital stay, site of care, laboratory diagnostics requested and type of treatment received) from the medical records. In addition, the resources consumed by each patient were identified and recorded using a Microsoft Excel 2010 spreadsheet. At Bokoji hospital Covid-19 treatment centre, all expenses related to Covid-19 cases management were provided free of charge and covered by the treatment centre. Therefore, the hospital/provider perspective was selected to determine the hospitalization costs of Covid-19 case management. The healthcare resources data on the type of staff and their salaries, allowances and duty payments, overhead (fuel, water, electricity, catering services, etc.), non-medical capital equipment and supply costs were collected from the hospital finance department. Data on drugs, medical capital equipment and medical supplies consumed were identified from the pharmacy department, whereas data on laboratory tests and diagnostic tests (e.g. X-ray and ultrasound) were retrieved from their respective hospital departments.

The costing analysis was carried out from the hospital perspective using both top-down and micro-costing approaches to estimate the hospitalisation costs of Covid-19 case managements. Direct costs, such as drugs, laboratory tests, diagnostic examinations and blood components,were estimated by multiplying the unit price of the services by the number of resources consumed based on data sources available in the hospital/treatment centre. Using a top-down costing methodology, the direct costs of medical supplies and personal protective equipment were allocated for each patient-day. To determine the total personnel costs, we summed the salary, allowances and duty time payments for all medical and non-medical staff. Then, the total personnel costs were apportioned by bed-day, resulting in daily personnel cost estimates for each disease severity. The costs of catering services were estimated using price data from the finance department multiplied by the length of stay(LOS) of each patient. For capital assets, we determined the equivalent annual costs using initial capital outlay over their lifetime. We used an expected lifetime of 30 years for buildings and 10 years for equipment. The capital costs were annualised using their respective useful lifetimes and a 3% discount rate [25]. To calculate the costs of the building, we measured the total building area for the provision of services in the treatment centre and allocated on the basis of the proportional size of rooms dedicated to each disease category. In addition, the annualised capital equipment costs were apportioned by bed-day to each disease severity. Moreover, other overhead costs, such as laundry, cleaning, fuel, electricity and water, were also apportioned by bed-day, resulting in daily general cost estimates for each disease type. The costs were recorded in Ethiopian birr and converted into US dollars (US$) using an exchange rate of 1US$=35.28 birr. All the costs were adjusted for inflation using the consumer price index of the year 2020 as a base-year cost.

Statistical analyses were conducted using STATA version 16 software. For continuous data, means with standard deviation(SD) or medians with Interquartile Range(IQR) were computed, whereas categorical variables were expressed as the number of cases and proportions. To check the cost data distribution, we employed the Shapiro-Wilk test. We used the t-test, Mann-Whitney U and Kruskal-Wallis tests to compare the differences in costs between various sociodemographic and clinical variables of patients. The relationships between hospitalisation costs and potential contributing factors were investigated using a multiple linear regression model. To express the proportion of hospitalisation expenditures, we transformed the hospitalisation costs by the natural logarithm and exponentiated the partial coefficients of the linear regression scaled by the natural log. A p-value of 0.05 or less was considered statistically significant.

## Results

### Sociodemographic characteristics of patients

A total of 692 confirmed Covid-19 patients were hospitalised,and their mean age was 40 ± 11.69 years. The greatest proportion of patients (35.7%) were in the age group of 18–35 years, and 446 (64.5%) were male. More than three-fifths (63.1%) of the hospitalised cases were from urban areas. The largest proportion (229;33.1%) of the cases were severe, followed by mild cases (151; 21.8%) and moderate cases (147; 21.2%). More than half (52.3%) the hospitalised cases stayed 15 or more days in the treatment centre, with a median LOS of 16 days (IQR: 10–23). About 27.7% of the respondents had comorbidities,and 55.6% of the participants received intranasal oxygen supplementation care (Table 1).

**Table 1:** Sociodemographic and clinical characteristics of participants admitted at Bokoji Hospital Covid-19 Treatment Centre in Ethiopia, 2021

Variables	Categories	Frequency (%)
Sex	Female	246 (35.5)
	Male	446 (64.5)
Age	< 18years	176 (25.4)
	18–35 years	247 (35.7)
	36–54 years	164 (23.7)
	≥ 55 years	105 (15.2)
	Mean(SD)	40 (11.69)
Residence	Rural	255 (36.9)
	Urban	437 (63.1)
Status at admission	Asymptomatic	138 (19.9)
	Mild	151 (21.8)
	Moderate	147 (21.3)
	Severe	229 (33.1)
	Critical	27 (3.9)
Comorbidity	Yes	192 (27.8)
	No	500 (72.2)
Place of admission	Ward	625 (90.3)
	ICU	27 (3.9)
	Both	40 (5.8)
Intranasal oxygen supplementation	Yes	385 (55.6)
	No	307 (44.4)
Length of stay	≤ 15 days	330 (47.7)
	> 15 days	363 (52.3)
Clinical outcome	Cured	629 (90.9)
	Died	63 (9.1)

### Cost per case treated

The total cost of hospitalisation for 692 admissions was US$956,690.00. The average cost per treated case was US$1,382.50 (SD: US$293.45), and the overall daily cost was US$82.12. Staff salaries account for the highest mean cost (64.1%), followed by the capital cost, which accounts for 21.2% of the overall cost. Total mean costs for supplies (masks, hand sanitiser, aprons, etc.) were US$41.99, accounting for 3.04% of the overall cost (Table 2).

**Table 2:** Average cost per treated case across different cost categories (2020 US$)

Cost categories	Mean cost per patient
Medications	$40.75
Laboratory & diagnostic examinations	$6.28
Catering services	$53.44
Personnel costs	$885.56
Capital costs	$292.58
Bed charges	$38.18
Supplies	$41.99
Utilities (water, electricity, fuel, etc.)	$23.72
Total cost	$1,382.50

Table 3 shows the factors that influence the hospitalisation costs of Covid-19 cases. There is significant variation in the total mean cost across various sociodemographic and clinical characteristics of hospitalised cases. Female patients are associated with a significantly higher hospitalisation cost compared to males (US$1,406.53 versus US$1,369.25, respectively; p < .001). We found higher mean hospitalisation costs in patients older than 55 years than in younger cases (US$1,721.42 versus US$1,154.67, respectively; p < .001). Cases with comorbidities had higher hospitalisation costs than cases without comorbidities(US$1,588.02 versus US$1,303.58, respectively;p < .001 ). In addition, patients who received intranasal oxygen care (p < .001), had severe disease (p < .001) and stayed for a long period on treatment (p < .001) were associated with higher mean hospitalisation costs (Table 3).

**Table 3:** Comparison of sociodemographic and clinical data and mean hospitalisation costs (2020 US$)

Variable	Average cost per case(US$)	P-value
Total	1,382.50 (293.45)	
Sex		
Female	1,406.53 (299.89)	0.027
Male	1,369.25 (289.33)	
Age		
< 18 years	1,154.67 (159.74)	< 0.001
18–35 years	1,329.65 (241.67)	
36–54 years	1,489.62 (249.27)	
≥ 55 years	1,721.42 (255.27)	
Disease severity		
Asymptomatic	1,073.86 (10.81)	0.001
Mild	1,100.74 (32.41)	
Moderate	1,394.74 (54.98)	
Severe	1,708.05 (160.91)	
Residence		
Rural	1,361.89 (272.66)	0.221
Urban	1,394.53 (304.59)	
Comorbidity		
No	1,303.58 (266.56)	< 0.001
Yes	1,588.02 (259.12)	
Place of admission		
Ward	1,335.10 (251.27)	< 0.001
ICU	2,127.36 (141.19)	
Both	1,620.35 (155.49)	
Intranasal oxygen used		
No	1,103.44 (70.25)	< 0.001
Yes	1,605.03 (197.79)	
Length of hospital stay		
≤ 15 days	1,176.43 (176.65)	0.001
> 15 days	1,570.36 (249.37)	

### Factors associated with hospitalisation costs

As the distribution of the total hospitalisation cost was skewed, we log-transformed the cost data before running the linear regression model. We exponentiated the coefficients of the total cost in the linear regression models to enable the interpretation of our findings (shown in Table 3). We found higher hospitalisation costs in ages 18–35 years (14%), ages 36–54 years (17%) and ages ≥ 55 years (25%) compared to ages under 18years.The costs of hospitalisation for cases with comorbidities were 25% higher than for those without underlying diseases. In addition, we found a 45% increase in hospitalisation costs among cases that received oxygen inhalation therapy over their counterparts. Furthermore, cases that were treated in both the ICU and the ward as well as in the ICU only were associated with increases of 23% and 62% in hospitalisation costs, respectively.The presence of signs of mild, moderate and severe symptoms during admission were associated with increases of 2%, 30% and 58% in hospitalisation costs, respectively. Moreover, cases hospitalised for 15–21 days and ≥ 22 days had 24% and 54% higher hospitalisation costs than those who stayed for less than 15 days in the treatment centre (Table 4).

**Table 4:** Multivariate linear regression analysis for factors associated with Covid-19 cost of hospitalisation in Bokoji Hospital, Ethiopia, 2021

Variables	Coefficient	SE	P-value	Exp (β)	95% CI
Age (versus < 18 years)					
18–35 years	0.124	0.018	0.001	1.14	1.09–1.18
36–54 years	0.226	0.022	0.001	1.17	1.11–1.23
≥ 55 years	0.157	0.026	0.001	1.25	1.20–1.31
Sex (ref: female)	−0.026	0.016	0.113	0.98	0.94–1.01
Residence (ref: rural)	0.021	0.017	0.215	1.02	0.99–1.06
Pre-existing disease (ref: no)	0.225	0.015	0.001	1.25	1.22–1.30
Intranasal oxygen use (ref: no)	0.369	0.008	0.001	1.45	1.42–1.47
Ward and ICU (ref: no)	0.206	0.030	0.001	1.23	1.16–1.30
ICU only (ref: no)	0.481	0.036	0.001	1.62	1.51–1.74
Mild (ref: asymptomatic)	0.024	0.007	0.001	1.02	1.01–1.04
Moderate (ref: mild)	0.261	0.008	0.001	1.30	1.27–1.32
Severe/critical (ref: mild)	0.460	0.006	0.001	1.58	1.56–1.61
15–21 days (ref: <14 days)	0.217	0.012	0.001	1.24	1.21–1.27
≥ 22 days (ref: <14 days)	0.434	0.016	0.001	1.54	1.49–1.60

Note: SE = standard error

## Discussion

In this study, we investigated the determinants of hospitalisation costs of Covid-19 casesat Bokoji Hospital Covid-19 Treatment Centre in South Central Ethiopia.The average hospitalisation cost was around US$1,382.50 (95% CI: 1,360.60–1,404.40). This finding is lower than the mean cost identified in studies done in Brazil (US$12,637.42) [26], Saudi Arabia(US$13,476) [27], the USA (US$24,826) [28],Portugal (€8,177) [29], Iran (US$3,755) [10] and Addis Ababa, Ethiopia (US$1,473) [30]. However, it is higher than findings of the studies done in China (US$1,177.81) [31] and Tehran, Iran (US$209.22) [32]. This may be due to differences in the method of quantifying, measuring and valuing costs as well as in disease severity status among hospitalised patients, which escalates the mean hospitalisation cost.

In the multivariate linear regression analysis, the factors that increased the hospitalisation cost were older age, presence of comorbidities, use of oxygen inhalation therapy, site of treatment, severe clinical conditions at admission andmore days of hospitalisation. We found older age was also independently associated with higher hospitalisation. This contradicts a report from Iran [33] but it is supported by reports from Brazil [26], the USA [34], China [31, 35] and Spain [36]. The higher cost in older cases was related to their higher risk of Covid-19 infection, morbidity and the presence of underlying diseases [37–40]. In addition, elderly patients have chronic illnesses that suppress their body’s immunity and are prone to develop a critical illness or even die [41, 42], which also increases their admission costs as a result of the need for advanced medical care to stabilise their condition.

In addition, we observed higher hospitalisation costs in cases treated with oxygen inhalation therapy as compared to their counterparts. This finding is supported by the findings from studies done in Brazil [26] and the USA [34]. This can be explained by the fact that the majority of patients who utilise inhalation therapy are severe and critical cases [27, 43] requiring specialised medical services to recover from the illness, which increases their hospitalisation cost. Comorbidities such as hypertension, diabetes, cardiovascular disease and respiratory disease were also independently associated with higher admission costs. This is in line with findings from studies conducted in Brazil [26], the USA [28, 34], China [35] and Iran [33]. This may be related to the fact that cases with underlying disease have severe conditions, and clinical aggravation during hospitalisation [44] that need specialised healthcare in the treatment of such cases, which is resource intensive and increases hospitalisation costs.

Patients who were admitted to the ICU were more likely to have higher hospitalisation costs than Covid-19–infected individuals who were admitted to the ward only. This finding is supported by the findings of studies conducted in the USA [34], Brazil [26] and Iran [32, 33]. In addition, patients with a prolonged LOS were more likely to have higher hospitalisation costs compared to Covid-19–infected individuals with a short LOS. The association between prolonged LOS, disease severity and pre-existing health conditions has been reported in previous studies [45–49]. Therefore, it is logical to assume that patients with prolonged LOS frequently have comorbidities, severe illness and complications. These factors raise the cost of hospitalisation by necessitating intensive care, expensive antiviral medications, inhalation oxygen therapy, non-invasive mechanical ventilation and more concentrated time from healthcare professionals.

### Limitations of the study

To the best of our knowledge, this is the first study to evaluateCovid-19 hospitalisation costs and their determinants in the Ethiopian setting. However, this study has several limitations. First, we conducted the study in a single facility, which may limit the generalisation of our results to other settings which have no similar set up. Second, we collected sociodemographic and clinical information from medical records,but other factors that are not available in medical records and may influence hospitalisation costs may have been missed. Third, this costing analysis included costs only from the healthcare provider’s perspective and therefore does not also indicate the economic burden of disease on patients and societies.

## Conclusions

According to this study, the average hospitalisation costs for Covid-19 are substantial. Factors such as older age, disease severity, presence of comorbidities, use of inhalation oxygen therapy and prolonged hospital stay are associated with higher hospitalisation costs. Therefore, the government should give priority to the elderly and those who have comorbidities in the provision of vaccination to reduce the financial burden on health facilities and health systems in terms of resource utilisation.

## Data Availability

The data sets used or analysed during this study are available from the corresponding author upon reasonable request.

## Abbreviations

GDP: gross domestic product
IQR: interquartile range
ICU: intensive care unit
LOS: length of stay
SARS-CoV2: severe acute respiratory syndrome coronavirus 2,SD:standard deviation
SE: standard error
USA: United State of America
US$: United States dollar
WHO: World Health Organization

## References

[1] WSDOH. Middle East respiratory syndrome coronavirus (MERS-CoV) infection. In: Washington, editor. 2017. 10.12968/hmed.2017.78.1.23 28067571

[2] Wiersinga WJ, Rhodes A, Cheng AC, Peacock JS (2020). Pathophysiology, transmission, diagnosis, and treatment of coronavirus disease 2019 (COVID-19):a review. JAMA.

[3] ECDC. An outbreak of acute respiratory syndrome associated with a novel coronavirus, China: first local transmission in the EU/EEA third update. Rapid risk Assessment. 2020.

[4] Unhale SS, Ansar QB, Sanap S, Thakhre S, Wadatkar S, Bairagi R (2020). A review on coronavirus (COVID-19). WJPLS.

[5] World Health Organization. Weekly operational update on COVID-19. 5 July 2021. https://www.who.int/publications/m/item/weekly-operational-update-on-covid-19.

[6] FMOH. Covid-19 daily situational analysis report. Report No.Addis Ababa; 14 March 2022.

[7] World Health Organization. WHO coronavirus (COVID-19) dashboard 2021. https://covid19.who.int/. Accessed 21April 2021.

[8] McKibbin W, Fernando R (2021). The global macroeconomic impacts of COVID-19: seven scenarios. Asian Economic Papers.

[9] Gondwe G. In:. Assessing the impact of COVID-19 on Africa’s economic development. United Nations Conference on Trade and Development; 2020.

[10] Ghaffari Darab M, Keshavarz K, Sadeghi E, Shahmohamadi J, Kavosi Z (2021). The economic burden of coronavirus disease 2019 (COVID-19): evidence from Iran. BMC Health Serv Res.

[11] Zikargae MH. COVID-19 in Ethiopia: assessment of how the ethiopian government has executed administrative actions and managed risk communications and community engagement. Risk Manage Healthc Policy. 2020:2803–10. 10.2147/RMHP.S278234 PMC7721303 33299368

[12] Tolu LB, Ezeh A, Feyissa GT. How prepared is Africa for the COVID-19 pandemic response? The case of Ethiopia. Risk Manage Healthc Policy. 2020:771–6. 10.2147/RMHP.S258273 PMC7358088 32753990

[13] Persad G, Pandya A (2022). A comprehensive Covid-19 response—the need for economic evaluation. N Engl J Med.

[14] Cirrincione L, Plescia F, Ledda C, Rapisarda V, Martorana D, Moldovan RE et al. COVID-19 pandemic: prevention and protection measures to be adopted at the workplace. Sustainability 2020;12(3603).

[15] Mboowa G, Musoke D, Bulafu B, Aruhomukama D (2021). Face-masking, an acceptable protective measure against COVID-19 in Ugandan high-risk groups. Am J Trop Med Hyg.

[16] Weiss BD, Paasche-Orlow MK. Disparities in adherence to COVID-19 public health recommendations. SLACK Incorporated Thorofare, NJ.2020;e171–3. 10.3928/24748307-20200723-01 PMC7410493 32926173

[17] Association AH. Hospitals and health systems face unprecedented financial pressures due to COVID-19. 2020.

[18] Cutler DM, Summers LH (2020). The COVID-19 pandemic and the $16 trillion virus. JAMA.

[19] Di Fusco M, Shea KM, Lin J, Nguyen JL, Angulo FJ, Benigno M (2021). Health outcomes and economic burden of hospitalized COVID-19 patients in the United States. J Med Econ.

[20] Kazempour-Dizaji M, Sheikhan F, Varahram M, Rouzbahani R, Vand MY, Khosravi B et al. Changes in a hospital’s costs and revenues before and after COVID-19: a case study of an iranian hospital. Health Scope. 2021;10(3).

[21] Alegbeleye BJ, Mohammed RK (2020). Challenges of healthcare delivery in the context of COVID-19 pandemic in sub-saharan Africa. Iberoamerican J Med.

[22] Emanuel EJ, Persad G, Upshur R, Thome B, Parker M, Glickman A, et al. Fair allocation of scarce medical resources in the time of Covid-19. New England Journal of Medicine; 2020. 10.1056/NEJMsb2005114 32202722

[23] Moodley K, Rennie S, Behets F, Obasa AE, Yemesi R, Ravez L (2021). Allocation of scarce resources in Africa during COVID-19: utility and justice for the bottom of the pyramid?. Dev World Bioeth.

[24] Ogunkola IO, Adebisi YA, Imo UF, Odey GO, Esu E, Lucero-Prisno DEIII (2021). Impact of COVID-19 pandemic on antenatal healthcare services in sub-saharan Africa. Public Health in Practice.

[25] Barasa E, Kairu A, Maritim M, Were V, Akech S, Mwangangi M (2021). Examining unit costs for COVID-19 case management in Kenya. BMJ Global Health.

[26] Miethke-Morais A, Cassenote A, Piva H, Tokunaga E, Cobello V, Gonçalves FAR et al. COVID-19-related hospital cost-outcome analysis: the impact of clinical and demographic factors. Brazilian J Infect Dis. 2021;25. 10.1016/j.bjid.2021.101609 PMC8373618 34454894

[27] Al Mutair A, Layqah L, Alhassan B, Alkhalifah S, Almossabeh M, AlSaleh T (2022). The estimated cost of treating hospitalized COVID-19 patients in Saudi Arabia. Sci Rep.

[28] Shrestha SS, Kompaniyets L, Grosse SD, Harris AM, Baggs J, Sircar K, et al. editors. Estimation of coronavirus disease 2019 hospitalization costs from a large electronic administrative discharge database, March 2020–July 2021. Open Forum Infectious Diseases. 2021. 10.1093/ofid/ofab561 PMC8686820 34938822

[29] Seringa J, Pedreiras S, Freitas MJ, de Matos RV, Rocha J, Millett C (2022). Direct costs of COVID-19 inpatient admissions in a portuguese tertiary care university centre. Portuguese J Public Health.

[30] Memirie ST, Yigezu A, Zewdie SA, Mirkuzie AH, Bolongaita S, Verguet S (2022). Hospitalization costs for COVID-19 in Ethiopia: empirical data and analysis from Addis Ababa’s largest dedicated treatment center. PLoS ONE.

[31] Li B, Chen L, Shi L (2022). Determinants of hospitalization costs among moderate cases of COVID-19. INQUIRY: The Journal of Health Care Organization Provision and Financing.

[32] Damiri S, Nahvijou A, Sargazi N, Fazaeli AA, Akbari Sari A, Daroudi R (2021). Hospitalization costs of patients with Covid-19: a study in Tehran University of Medical Sciences. Health Manage Inform Sci.

[33] Ramezani-Doroh V, Tapak L, Hamidi Y, Bashirian S, Soltanian AR, Motaghed M (2022). Which patients bring the most costs for the hospital? A study on the cost determinants among COVID-19 patients in Iran. Cost Eff Resource Allocation.

[34] Ohsfeldt RL, Choong CK-C, McCollam PL, Abedtash H, Kelton KA, Burge R (2021). Inpatient hospital costs for COVID-19 patients in the United States. Adv Therapy.

[35] Dong M, Yang Z, Chen Y, Sun J, Ma W, Cheng S (2021). Hospitalization costs of COVID-19 cases and their associated factors in Guangdong, China: a cross-sectional study. Front Med.

[36] Drago G, Pérez-Sádaba FJ, Aceituno S, Gari C, López-Belmonte JL (2023). Healthcare resource use and associated costs in a cohort of hospitalized COVID-19 patients in Spain: a retrospective analysis from the first to the third pandemic wave. EPICOV study. PLoS ONE.

[37] Rozenfeld Y, Beam J, Maier H, Haggerson W, Boudreau K, Carlson J (2020). A model of disparities: risk factors associated with COVID-19 infection. Int J Equity Health.

[38] Pijls BG, Jolani S, Atherley A, Derckx RT, Dijkstra JI, Franssen GH (2021). Demographic risk factors for COVID-19 infection, severity, ICU admission, and death: a meta-analysis of 59 studies. BMJ Open.

[39] Parohan M, Yaghoubi S, Seraji A, Javanbakht MH, Sarraf P, Djalali M (2020). Risk factors for mortality in patients with coronavirus disease 2019 (COVID-19) infection: a systematic review and meta-analysis of observational studies. The Aging Male.

[40] Bonanad C, García-Blas S, Tarazona-Santabalbina F, Sanchis J, Bertomeu-González V, Fácila L (2020). The effect of age on mortality in patients with COVID-19: a meta-analysis with 611,583 subjects. J Am Med Dir Assoc.

[41] Honardoost M, Janani L, Aghili R, Emami Z, Khamseh ME (2021). The association between the presence of comorbidities and COVID-19 severity: a systematic review and meta-analysis. Cerebrovasc Dis.

[42] Ji W, Huh K, Kang M, Hong J, Bae GH, Lee R et al. Effect of underlying comorbidities on the infection and severity of COVID-19 in Korea: a nationwide case-control study. J Korean Med Sci. 2020;35(25). 10.3346/jkms.2020.35.e237 PMC7324262 32597048

[43] Ferrario L, Schettini F, Porazzi E, Dalla Bona M, Pagani M, Dezi T (2022). POSB103 COVID-19 positive patients hospital management in the italian setting: the economic impact. Value in Health.

[44] Terada M, Ohtsu H, Saito S, Hayakawa K, Tsuzuki S, Asai Y (2021). Risk factors for severity on admission and the disease progression during hospitalization in a large cohort of patients with COVID-19 in Japan. BMJ Open.

[45] Crankson S, Pokhrel S, Anokye NK (2022). Determinants of COVID-19–related length of hospital stays and long COVID in Ghana: a cross-sectional analysis. Int J Environ Res Public Health.

[46] Thai PQ, Toan DTT, Son DT, Van HTH, Minh LN, Hung LX et al. Factors associated with the duration of hospitalization among COVID-19 patients in Vietnam: a survival analysis. Epidemiol Infect.148(e114):1–7. 10.1017/S0950268820001259 PMC7306545 32517822

[47] Abrahim SA, Tessema M, Defar A, Hussen A, Ejeta E, Demoz G et al. Time to recovery and its predictors among adults hospitalized with COVID-19: a prospective cohort study in Ethiopia. PLoS ONE. 2020;15(12). 10.1371/journal.pone.0244269 PMC7773180 33378367

[48] Liu X, Zhou H, Zhou Y, Wu X, Zhao Y, Lu Y (2020). Risk factors associated with disease severity and length of hospital stay in COVID-19 patients. J Infect.

[49] Alwafi H, Naser AY, Qanash S, Brinji AS, Ghazawi MA, Alotaibi B (2021). Predictors of length of hospital stay, mortality, and outcomes among hospitalised COVID-19 patients in Saudi Arabia: a cross-sectional study. J Multidisciplinary Healthc.

